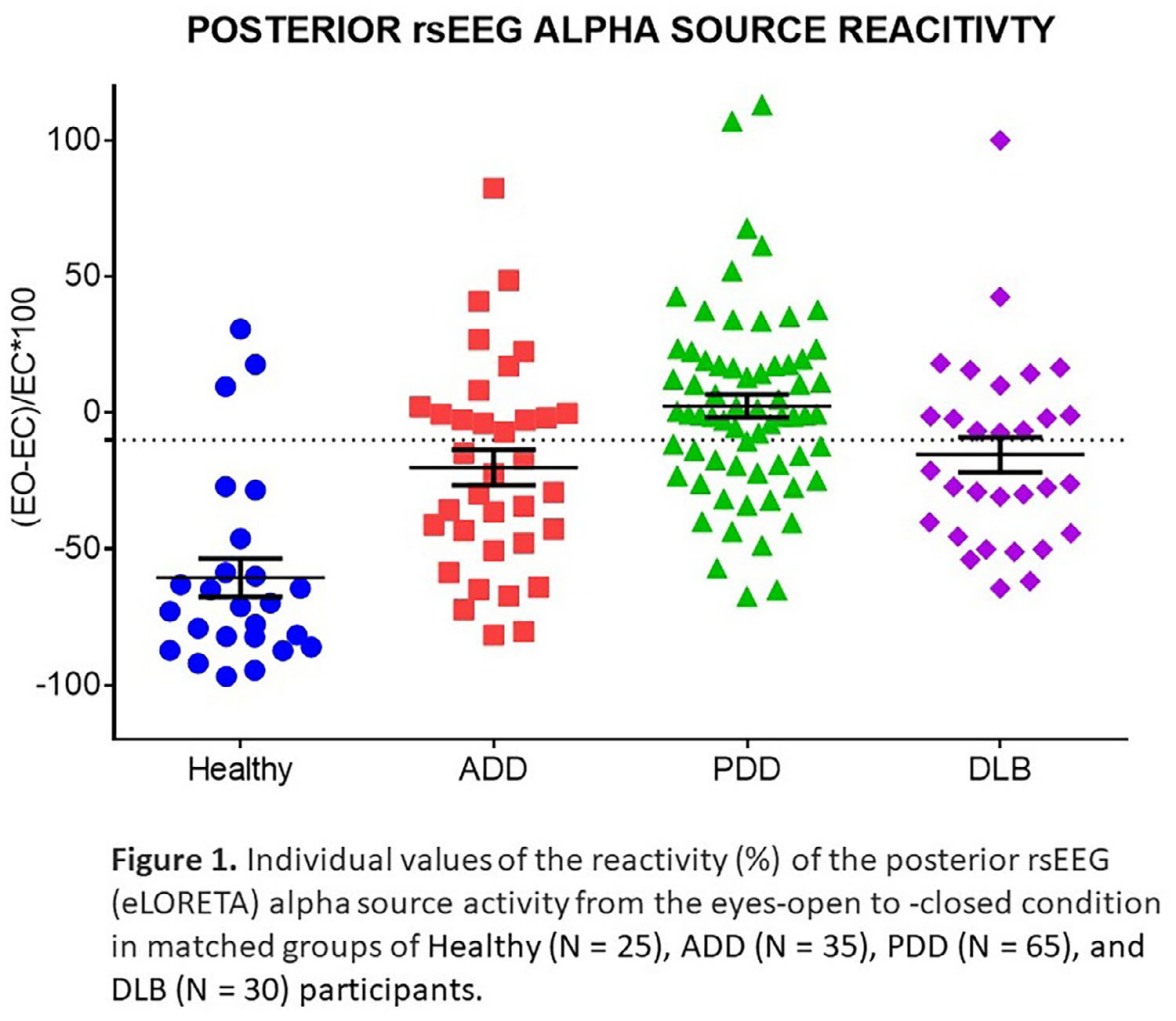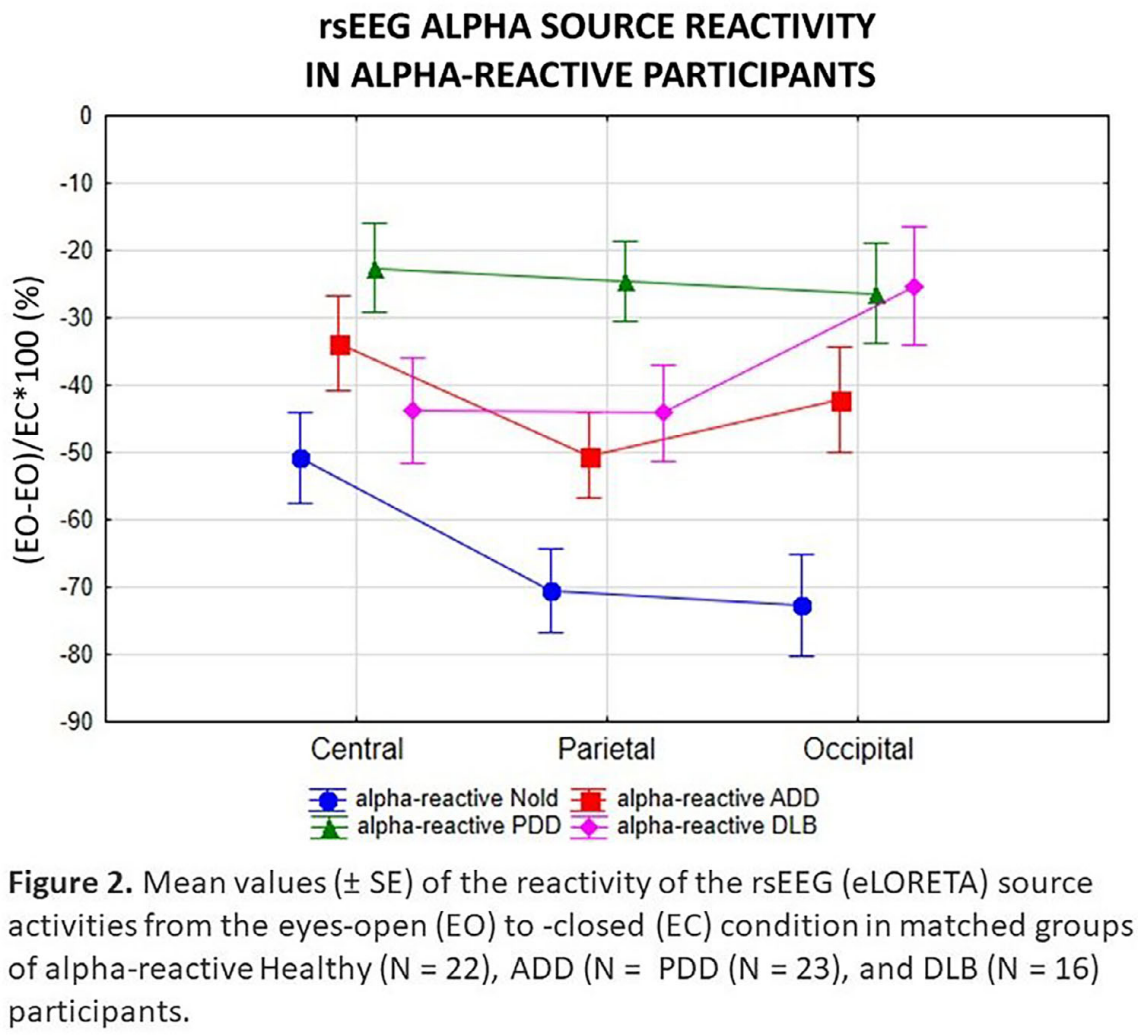# Underlying mechanisms for altered reactivity of posterior cortical electroencephalographic alpha rhythms in DLB and PDD

**DOI:** 10.1002/alz.085690

**Published:** 2025-01-09

**Authors:** Roberta Lizio, Claudio Del Percio, Giuseppe Noce, Susanna Lopez, Dharmendra Jakhar, Matteo Carpi, Dario Arnaldi, Bahar Güntekin, Görsev Yener, Raffaele Ferri, Giovanni B. Frisoni, Claudio Babiloni

**Affiliations:** ^1^ Supervisor and Early Stage Researcher’s project leader in the Blood Biomarker‐based Diagnostic Tools for Early Stage Alzheimer’s Disease (BBDiag) project (HORIZON 2020 Marie Skłodowska‐Curie MSCA‐ITN‐ETN; 721281; 2017‐2020, Rome Italy; ^2^ IRCCS SDN, Naples Italy; ^3^ Sapienza University of Rome, Rome Italy; ^4^ Sapienza University of Rome, Rome, Rome Italy; ^5^ IRCCS Synlab SDN, Naples Italy; ^6^ IRCCS Ospedale Policlinico San Martino, Genoa Italy; ^7^ University of Genoa, Genoa Italy; ^8^ Istanbul Medipol University, Istanbul Turkey; ^9^ Izmir University of Economics, Faculty of Medicine, Balçova, Izmir Turkey; ^10^ Oasi Research Institute ‐ IRCCS, Troina Italy; ^11^ University of Geneva, Geneva Switzerland; ^12^ IRCCS Istituto Centro San Giovanni di Dio Fatebenefratelli, Brescia Italy

## Abstract

**Background:**

The amplitude of resting‐state electroencephalographic (rsEEG) rhythms is a promising neurophysiological biomarker to investigate the abnormalities of oscillatory neurophysiological thalamocortical mechanisms related to the general cortical arousal and vigilance in wakefulness in patients with dementia due to neurodegenerative diseases as Alzheimer’s disease (ADD), Parkinson’s disease (PDD) and Lewy Body disease (DLB). Here, we tested the hypothesis that the reactivity of posterior rsEEG alpha (about 8‐12 Hz) rhythms during the transition from eyes‐closed to ‐open condition may be lower in PDD patients than in DLB patients.

**Methods:**

A Eurasian database provided clinical‐demographic‐rsEEG datasets in 35 ADD patients, 65 PDD patients, 30 DLB patients, and 25 matched cognitively unimpaired (Healthy) persons. The rsEEG rhythms were investigated at individual delta, theta, and alpha frequency bands, as well as fixed beta (14‐30 Hz) and gamma (30‐40 Hz) bands. The eLORETA freeware was used to estimate cortical rsEEG sources.

**Results:**

Results showed substantial (i.e., greater than ‐10%) reduction (reactivity) in the posterior alpha source activities from the eyes‐closed to the eyes‐open condition in 88% of the Healthy seniors, 53% of ADD patients, 53% of the DLB patients, and only 37% of the PDD patients (Figure 1). In these alpha‐reactive participants, there was lower reactivity in the posterior alpha source activities in the three neurodegenerative (i.e., ADD, PDD, and DLB) groups than in the Healthy group. Furthermore, the reduction of the occipital alpha reactivity was stronger in the PDD and DLB groups than in the ADD group. Finally, the reduction of the parietal alpha reactivity was stronger in the PDD group than in the ADD and DLB groups (Figure 2).

**Conclusion:**

These results suggest that ADD, PDD, and DLB patients may be characterized by very poor reactivity in the posterior cortical mechanisms desynchronizing rsEEG alpha rhythms in relation to increased vigilance levels as an interesting neurophysiological biomarker. This biomarker may be used as a primary endpoint for interventions with drugs or brain electromagnetic stimulations to improve vigilance regulation and quality of life in neurodegenerative patients, allowing them to follow TV programs and interactions in their social environment.